# C/EBPα-p30 protein induces expression of the oncogenic long non-coding RNA UCA1 in acute myeloid leukemia

**DOI:** 10.18632/oncotarget.4069

**Published:** 2015-05-25

**Authors:** James M. Hughes, Ivano Legnini, Beatrice Salvatori, Silvia Masciarelli, Marcella Marchioni, Francesco Fazi, Mariangela Morlando, Irene Bozzoni, Alessandro Fatica

**Affiliations:** ^1^ Department of Biology and Biotechnology “C. Darwin”, Sapienza University of Rome, Rome, Italy; ^2^ Department of Anatomical, Histological, Forensic & Orthopaedic Sciences, Sapienza University of Rome, Rome, Italy; ^3^ Institute of Biology, Molecular Medicine and Nanobiotechnology, CNR, Sapienza University of Rome, Rome, Italy; ^4^ Center for Life Nano Science@Sapienza, Istituto Italiano di Tecnologia, Rome, Italy; ^5^ Institute Pasteur Fondazione Cenci-Bolognetti, Sapienza University of Rome, Rome, Italy; ^6^ Department of Systems Biology, Herbert Irving Comprehensive Cancer Center, Columbia University Medical Center, New York, NY, USA

**Keywords:** long non-coding RNA, acute myeloid leukemia, CEBPA, UCA1

## Abstract

Accumulating evidences indicate that different long non-coding RNAs (lncRNAs) might play a relevant role in tumorigenesis, with their expression and function already associated to cancer development and progression. *CCAAT/enhancer-binding protein-α* (CEBPA) is a critical regulator of myeloid differentiation whose inactivation contributes to the development of acute myeloid leukemia (AML). Mutations in C/EBPα occur in around 10% of AML cases, leading to the expression of a 30-kDa dominant negative isoform (C/EBPα-p30). In this study, we identified the oncogenic *urothelial carcinoma associated 1* (UCA1) lncRNA as a novel target of the C/EBPα-p30. We show that wild-type C/EBPα and C/EBPα-p30 isoform can bind the *UCA1* promoter but have opposite effects on UCA1 expression. While wild-type C/EBPα represses, C/EBPα-p30 can induce *UCA1* transcription. Notably, we also show that UCA1 expression increases in cytogenetically normal AML cases carrying biallelic *CEBPA* mutations. Furthermore, we demonstrate that UCA1 sustains proliferation of AML cells by repressing the expression of the cell cycle regulator p27^kip1^. Thus, we identified, for the first time, an oncogenic lncRNA functioning in concert with the dominant negative isoform of C/EBPα in AML.

## INTRODUCTION

Blocks in genetic programs required for terminal myeloid differentiation and aberrant proliferation characterize acute myeloid leukemia (AML) (reviewed in [1]). One of the key components that have been shown to be crucial for myeloid differentiation is the transcription factor CCAAT/enhancer-binding protein-*α* (C/EBPα) (reviewed in [2]). The intronless *CEBPA* mRNA can produce two translation products by using two different start codons within the same open reading frame: the full-length C/EBPα (p42) and a shorter form (p30) (reviewed in [3]). P30 isoform retains the DNA-binding domain, but lacks the N-terminal transactivation domain and exhibits a dominant-negative function over p42 isoform. Furthermore, independent or different p30 functions than that of p42 have been described in transcriptional regulation indicating that this isoform is more than just a dominant-negative regulator of p42 [4–7]. Specifically, p30 was shown to act as transcriptional activator of the PIN1 [6] and miR-181a [7] genes. The ratio of p30/p42 is critical for granulopoiesis as the p30 protein fails to induce differentiation and increases cell proliferation of myeloid progenitors [3, 8]. Relative levels of the two isoforms are controlled at the translational level to respond to extracellular conditions [3]. In AML, C/EBPα function is frequently disrupted by mutations in its locus or by the action of leukaemogenic fusion proteins [2, 3, 9]. In particular, approximately 10% of AML patients show dominant-negative mutations in the *CEBPA* coding region that in most of the cases abolish translation of full-length C/EBPα, leading to overexpression of the shorter p30 isoform [3, 8]. Notably, mouse models have shown that it is the increased expression of the p30 isoform, and not the loss of wild type C/EBPα, to be required for leukemia transformation [10]. Various downstream effectors have been shown to contribute to C/EBPα p42 and p30 activity, including non-coding RNAs (ncRNAs). At present, the majority of the studies have mainly focused on microRNAs [11–14]. These investigations showed that deregulation of specific C/EBPα-regulated microRNAs plays a critical role in leukemia initiation and outcome prediction. However, current studies on human transcriptome have shown that a relevant part of the genome is transcribed in the heterogeneous class of long non-coding RNAs (lncRNAs). Similarly to microRNAs, lncRNAs may have important functions in regulatory networks controlling diverse cellular processes, with their expression already associated with disorders, including cancer [15–17]. Thus, studying the expression and function of lncRNAs could help our understanding of leukemogenenis and in the identification of novel therapeutic targets. Different lncRNAs have been identified in normal and malignant hematopoiesis [18]. However, only a few have been identified and functionally characterized in the context of the AML-associated aberrant transcriptional programs [19, 20].

We therefore performed a genome-wide transcriptome analysis by RNA-sequencing to identify lncRNAs specifically regulated by the p30 isoform of C/EBPα. Among them, we identified the oncogenic *urothelial carcinoma associated 1* (UCA1) lncRNA [21–24]. We showed that both wild-type C/EBPα and C/EBPα-p30 isoforms can bind the *UCA1* promoter but have opposite effects on UCA1 expression. While wild-type C/EBPα repressed, C/EBPα-p30 strongly induced UCA1 transcription. Furthermore, we demonstrated that UCA1 sustains proliferation of AML cells by repressing the expression of the cell cycle regulator p27^kip1^. Notably, we also show that UCA1 expression is increased in primary cells from cytogenetically normal AML carrying biallelic CEBPA mutations. Therein, our findings indicate UCA1 as a novel diagnostic biomarker and a potential target for AML with CEBPA mutations.

## RESULTS

### *UCA1* transcripts is specifically induced by C/EBPα-p30 and up-regulated in AML with *CEBPA* mutations

**Figure 1:**
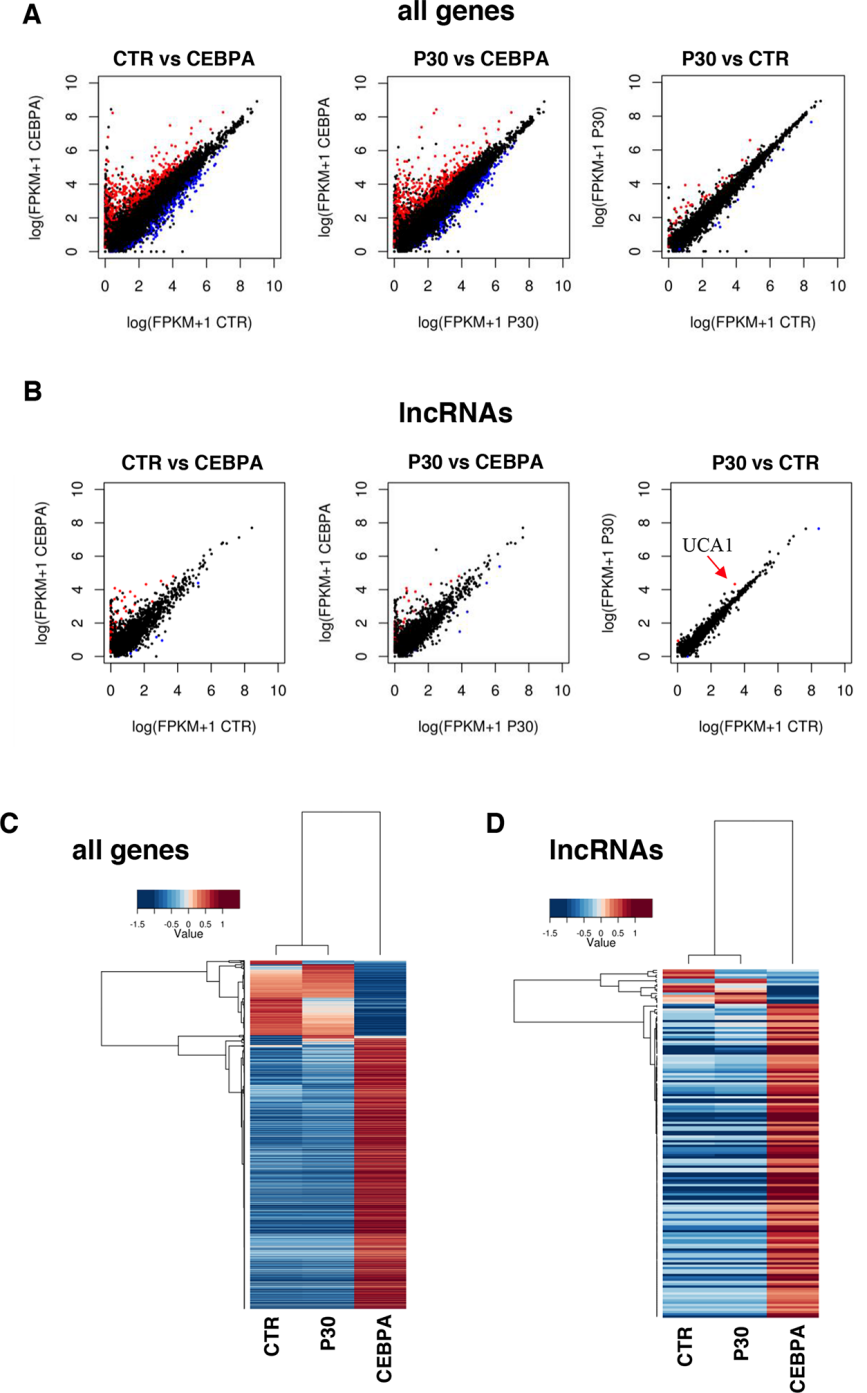
Global transcriptome profiling via RNA-Seq **A.** Pair-wise sample comparisons of differentially expressed coding genes, with red being significantly up-regulated and blue being significantly down-regulated. **B.** Pair-wise sample comparisons of differentially expressed lncRNAs, color coded the same as (A) An arrow indicates UCA1. **C.** This heatmap shows coding and non-coding genes significantly up-regulated or down-regulated between CTR, CEBPA and P30 cells as determined by CuffDiff, reported as fold change over mean. **D.** This heatmap shows differentially expressed lncRNAs as determined by CuffDiff, reported as fold change over mean.

**Figure 2:**
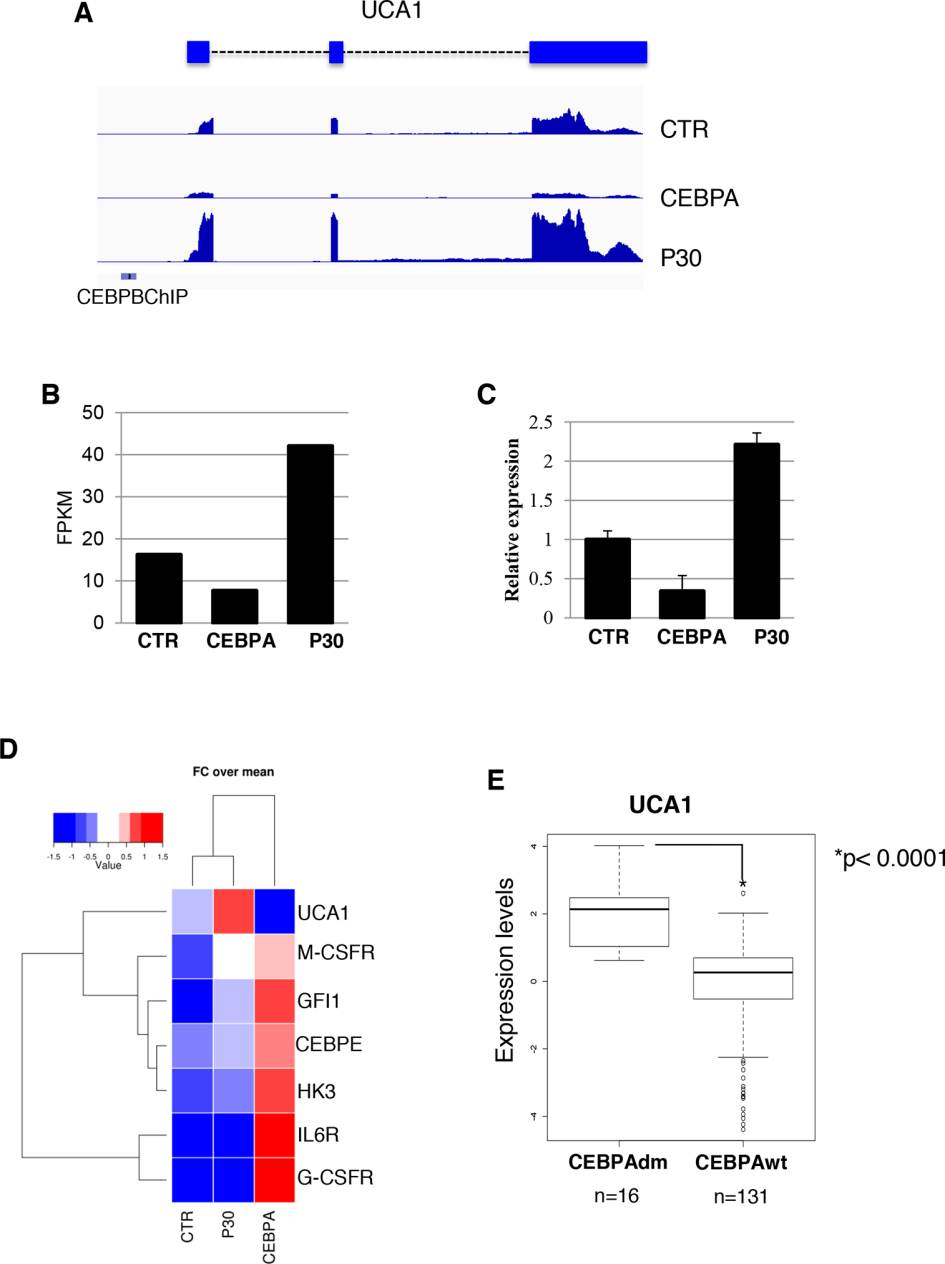
UCA1 expression levels in K562 cell lines and AML patients with CEBPA mutations **A.** RNA-Seq read alignment of UCA1 is displayed. Previously identified CEBPB ChIP-Seq binding sites from ENCODE in K562 are depicted with a blue bar. **B.** FPKM values report from Cuffdiff differential gene expression analysis. **C.** Analysis of UCA1 expression levels amongst the CTR, CEBPA and P30 samples by qRT-PCR. **D.** Heatmap showing fold change over mean values of UCA1 along with other known transcription targets of C/EBPα. **E.** Quantitative expression analysis of UCA1 in primary leukemia cells derived from AML patients with biallelic CEBPA mutations (CEBPAdm) or wild-type CEBPA (CEBPA-wt). *P* value is indicated.

To investigate the regulation of lncRNAs by the p30 dominant negative isoform of C/EBPα, we utilized the leukemic K562 cells, which do not express neither of the two C/EBPα isoforms, stably transformed with a PiggyBac transposon system [25] carrying Tet-inducible wild-type C/EBPα (CEBPA) or C/EBPα-p30 (P30) isoform. As expected, doxycycline (Dox) treatment of CEBPA cells restored C/EBPα expression and induced proliferation arrest and granulocytic differentiation ([25–27], while the Dox treatment on control empty vector (CTR) cells and P30 cells had no effect on proliferation (Supplementary Information, Figure S1). RNA-seq analysis was performed to search for lncRNAs that might be differentially regulated by the two C/EBPα isoforms. RNAs were analyzed by strand-specific and paired-end deep sequencing from biological duplicates. The total number of sequenced reads ranged from 50–70 million pairs for sample with similar percentage of alignment. Statistical analysis confirmed a near 95% correlation between replicates (Supplementary Information, Figure S2). Transcripts were mapped and assembled to the human genome (hg19) using TopHat and Cufflinks [28], respectively. Cuffdiff [29] was used to test for differential expression on Cufflinks assembled transcripts. A false discovery rate of 0.1 was used to select bona fide statistically significant changes in expression levels. We used the scale “log (FPKM+1)” to graph on a log scale and account for genes that had an FPKM of zero in one of the samples. The analysis revealed a total of 1079 protein coding RNAs and 170 lncRNAs in CTR vs CEBPA, 55 protein coding RNAs and 6 lncRNAs in CTR vs P30 and 949 protein coding RNAs and 155 lncRNAs in P30 vs CEBPA (Supplementary Information, Tables S1–S3). Significantly differentially expressed genes are showed in scatter plots (Figure 1A). A summary of all RNA biotypes used for this analysis can be seen in Supplementary Information, Figure S2. An unsupervised hierarchical clustering analysis on significant coding and non-coding transcripts with FPKM greater than 1 in either samples detected significant differences in expression between the three samples (Figure 1C). CTR vs CEBPA and P30 vs CEBPA were very similar and had almost identical distributions of protein coding and lncRNAs. This result reflects how the p30 isoform is inactive compared to wild type C/EBPα in transcriptional activity. Nevertheless, specific transcripts are modulated by the p30 isoform. We focused our analyses on the differentially expressed lncRNAs (Figures 1B and 1D). Interestingly, CTR vs P30 produced few significant changes in lncRNA expression. Among them, we noticed up-regulation of the *urothelial carcinoma associated 1* (*UCA1*), a well-known oncogenic lncRNA specifically induced in different types of solid tumors [21–24]. Our sequencing identified a single 2.3 kb UCA1 isoform, containing three exons, specifically up-regulated by the p30 isoform and down-regulated by the wild type C/EBPα (Figures 2A and 2B). Real-time PCR analysis (qRT-PCR) confirmed that, with respect to control cells, higher levels of UCA1 expression were detected in P30 cells and decreased expression in CEBPA (Figure 2C). Notably, UCA1 has opposite expression of well-known transcriptional targets of C/EBPα [25], confirming that our RNA-Seq data accurately reflect the activity of C/EBPα isoforms (Figure 2D).

To evaluate the significance of *UCA1* in AML, we evaluated its expression in a public gene expression profiling by array from a cohort of cytogenetically normal AML patients containing 16 AML cases with biallelic CEBPA mutations (CEBPAdm) and 130 AML cases with wild type CEBPA [30]. In patients with CEBPAdm, one allele usually contains a mutation in the DNA-binding domain, whereas the other contains the mutation producing the p30 isoform [3]. Consistent with the data in K562 cell lines, there was a positive and significant correlation between *UCA1* expression levels and CEBPA mutations (Figure 2E). While our studies were in progress, Bloomfield and co-workers reported a profiling of lncRNAs in cytogenetically normal AML cells carrying mutations in the NPM1, CEBPA, IDH2, ASXL1, and RUNX1 genes and internal tandem duplications in the FLT3 gene [31]. Notably, in agreement with our study, UCA1 expression was found specifically induced in patients carrying CEBPA mutations. Taken together, these finding indicate that mutations in CEBPA can induce UCA1 expression in AML.

### C/EBPα wild type and C/EBPα-p30 isoforms have opposite effect on UCA1 transcription

It has been previously shown that the *UCA1* promoter contains C/EBPα binding sites and that the C/EBPα protein can bind to the UCA1 promoter region both *in vitro* and *in vivo* in bladder cancer cells. However, in contrast of what we found in AML, C/EBPα seems to regulate UCA1 in an opposite manner [32]. In addition, ENCODE Chromatin immunoprecipitation (ChIP) data sets (accession ENCSR000EHE) showed binding of C/EBPβ, which exhibit identical DNA-binding specificities with C/EBPα [33] in the *UCA1* promoter region in K562 cells. Thus, to analyze the transcriptional regulation of UCA1 expression by C/EBPα isoforms, we performed ChIP and promoter-luciferase assays in K562 cells.

**Figure 3:**
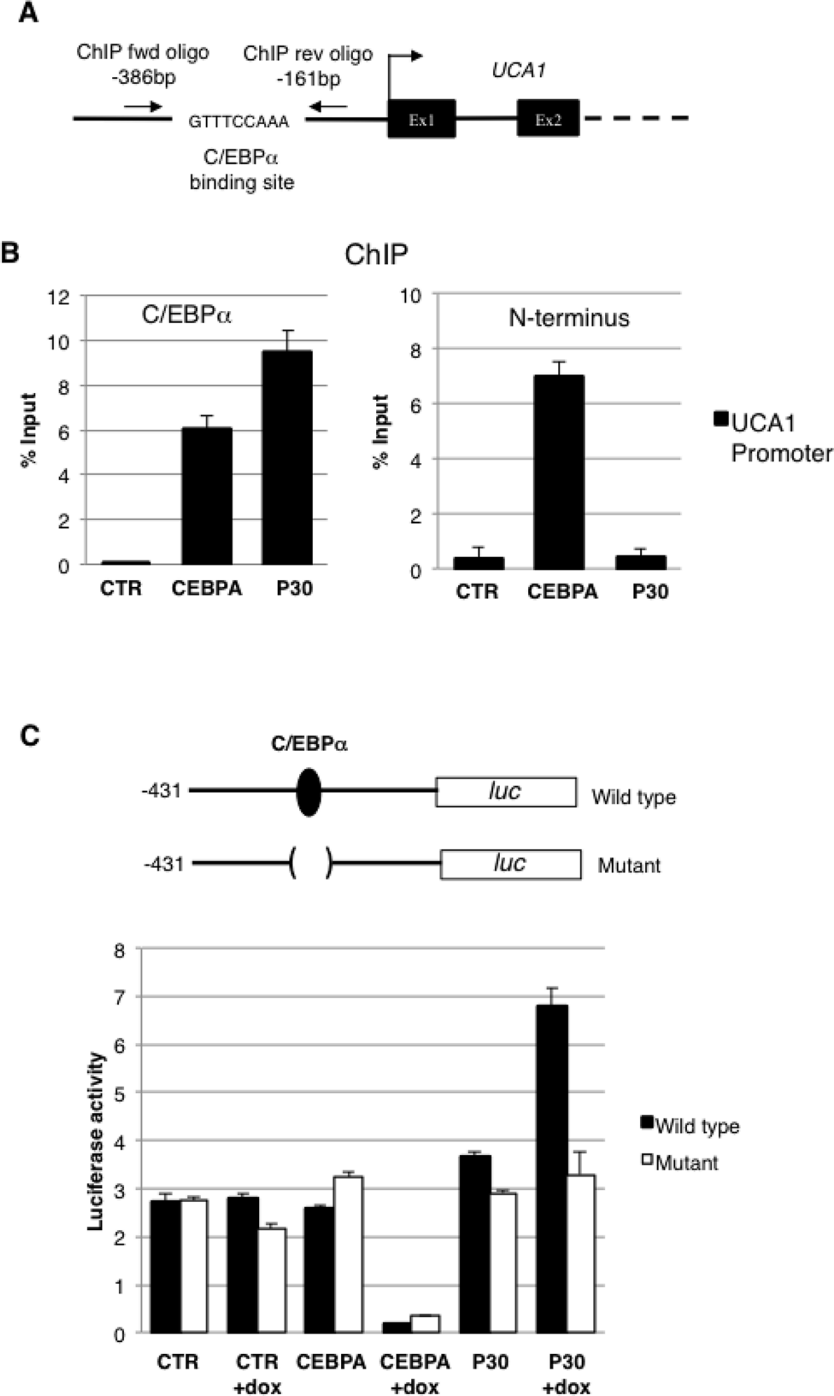
C/EBPα-p30 regulates UCA1 expression **A.** Schematic representation of the UCA1 promoter region, the putative C/EBPα binding site and oligonucleotides utilized in ChIP experiments are indicated. **B.** Chromatin from K562 cells was immunoprecipitated with anti-CEBPA antibodies recognizing both isoforms (left panel) or only the p42 (right panel), and the recovered DNA was quantified by real-time PCR. Results are expressed as the relative level over control cells after correcting for differences in the amount of starting (input) chromatin materials. The histograms represent the means ± S.E.M from three independent experiments. Binding sites for CEBPA and position of the qRT-PCR amplicon are indicated in the schematic representation. **C.** Schematic representation of the UCA1 promoter region and its mutant derivative in the putative C/EBPα binding site cloned in the pGL4 luciferase reporter vector. CTR, CEBPA, and P30 cells were transfected with UCA1 wild type and mutant promoter reporters. FF luciferase values were normalized to RL luciferase reading and then normalized again to empty pGL4 vector to obtain the transcription efficiency. The histograms represent the means ± S.E.M. from three independent experiments.

**Figure 4:**
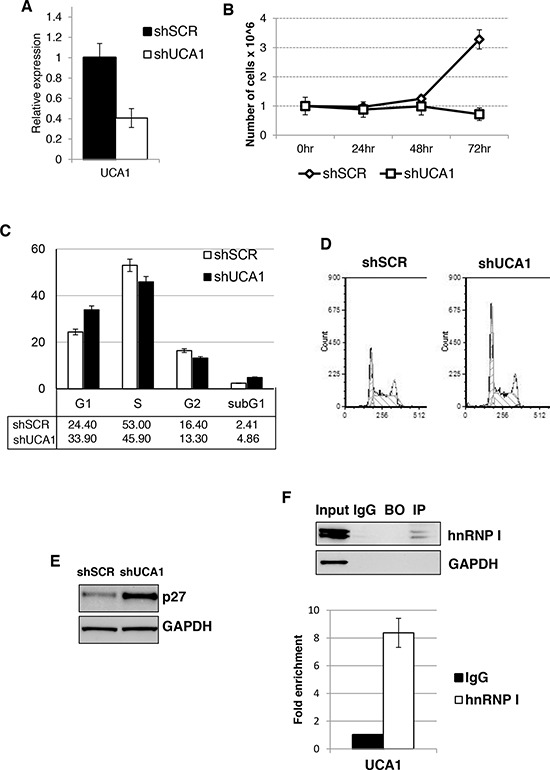
UCA1 sustain AML cell proliferation and regulated p27^kip1^ expression **A.** Effectiveness of UCA1 silencing in K562 cells. Level of expression was measure by qRT-PCR relative to HPRT mRNA. Error bars represent S.E.M., from three independent experiments, *P* < 0.05. **B.** Effect of shUCA1 and shSCR on cell proliferation. **C.** Cell cycle distribution of cells transduced with shUCA1 and shSCR lentivirus. The histograms represent the means ± S.E.M. from three independent experiments. **D.** Representative cell cycle analysis of (C) **E.** Western blot analysis of p27^kip1^ protein levels from cells transduced with shUCA1 and shSCR lentivirus. GAPDH was utilized as loading control. **F.** RIP was performed on cytoplasmic extracts from K562 cells. The extracts were incubated with anti-hnRNP I (IP) or control antibodies (IgG). Beads-only were used as control (BO). The eluted RNA was analyzed by qRT-PCR. IP efficiency was assessed by western blot.

To investigate whether C/EBPα isoforms physically interact *in vivo* with the *UCA1* promoter, we performed ChIP on CTR-, CEBPA- and P30 -K562 cells with an anti-C/EBPα antibody that recognized both isoforms (Figure 3). DNA from the immunoprecipitates was amplified with a couple of PCR primers located in the promoter region surrounding the C/EBPα binding sites (Figure 4A; [24]) and normalised to the amplification obtained with oligonucleotides corresponding to an unrelated genomic region. Control IgGs were utilized as second negative control. Immunoprecipitation on UCA1 promoters were detected with both wild-type C/EBPα and C/EBPα-p30 proteins while no signal was detected in CTR cells (Figures 3A and 3B). As expected, both C/EBPα isoforms can bind the UCA1 promoter since they share the same DNA binding capability [3]. ChIP performed with an antibody raised against the N-terminus of C/EBPα, which specifically recognized the p42 isoform, confirmed that the full-length C/EBPα proteins is able to bind the UCA1 promoter (Figure 3B).

Furthermore, to assess the contribution of C/EBPα and C/EBPα p30 isoforms on UCA1 transcription we constructed promoter-luciferase fusion constructs containing the wild-type UCA1 promoter or a mutant derivative lacking the putative C/EBPα binding site and tested the resulting luciferase activity in K562 cells expressing C/EBPα or the individual p30 isoform (Figure 3C). After transfection, cells were treated with Dox for 48 hours to allow protein expression and luciferase activity was measured. Despite both isoforms being able to bind the UCA1 promoter, only the p30 was able to induce its transcription, whereas C/EBPα showed repressing activity. Moreover, a strong reduction in p30-mediated transcriptional activation was detected with the mutant luciferase-reporter in the C/EBPα binding site. Notably, wild-type C/EBPα continued to repress transcription of the mutant promoter. These results correlate with the RNA-seq and qRT-PCR data showing reduced UCA1 expression in C/EBPα expressing cells compared to CTR cells (see Figures 2B and 2C). This might be explained by the activity of a transcriptional repressor induced by C/EBPα and not by p30 mutant that acts independently from C/EBPα protein on UCA1 promoter or by a differential binding of C/EBPα and p30 proteins on UCA1 promoter region. Taken together, these finding indicate that C/EBPα-p30 can specifically induce UCA1 transcription through direct binding in its promoter region.

### Knockdown of UCA1 decreases AML cell proliferation potential by inducing p27^kip1^ expression

As UCA1 was shown to promote cell growth in different solid tumors [21, 34, 35], we then analyzed the pro-proliferation activity of *UCA1* in AML cells. To do this, we utilized lentivirus infection to deliver short-hairpin RNA targeting UCA1 (shUCA1) alongside a control short-hairpin scramble sequence (shSCR) in our K562 background cell line. We obtained about 60% reduction of UCA1 levels with shUCA1 that resulted in significant reduction of proliferation (Figures 4A and 4B). Cell cycle analysis revealed an increase in the G1 cell population and decrease in the S phase cell population upon shUCA1 treatment (Figures 4C and 4D). Therein indicating that, similarly to other cancer cells, UCA1 is able to promote proliferation of AML cells.

After observing this block in proliferation and arrest in cell cycle progression, we sought to understand the mechanism by which UCA1 exerts its effects. One of the proposed mechanisms for UCA1 function was its capability to sequester hnRNP I protein, a positive translation regulator of the CDK inhibitor p27^kip1^, thus resulting in increased proliferation rate [23]. Therein, we analyzed p27^kip1^ protein levels after UCA1 knock-down and observed an increase in p27^kip1^ protein levels in the shUCA1 cells when compared to the control shSCR cell line (Figure 4E). Next, we performed an RNA immunoprecipitation (RIP) using antibodies for hnRNP I and probing for UCA1 by qRT-PCR (Figure 4F). Notably, we observed a significant increase of UCA1 levels with hnRNP I antibodies over IgG background levels, indicating an interaction of UCA1 and hnRNP I in K562 cells. Altogether these data indicate that UCA1 can contribute to the proliferation potential of AML cells by inhibiting p27^kip1^ expression.

## DISCUSSION

The last decade has seen exciting developments in the field of lncRNAs, suggesting that the involvement of lncRNAs in human diseases could be far more prevalent than previously appreciated [15–17]. Different studies have shown that changes in lncRNA expression may contribute to the development of cancer ([18–19, 36–39]. It follows that identification of the molecular mechanisms that regulate lncRNAs is imperative to the understanding of the molecular mechanisms involved in tumorigenesis and in the future development of novel therapies aimed at targeting cancer cells.

Mutations in the *CEBPA* gene, encoding the C/EBPα transcription factor, are observed in around 10% of AML cases and typically involve mutation of both *CEBPA* alleles [3]. The most common *CEBPA* mutation found in patients produces a short dominant negative isoform referred to as p30. P30 can regulate unique set of target genes and is more than a dominant-negative regulator of C/EBPα function [4–7]. Various downstream effectors have been shown to contribute to C/EBPα p30 activity, including non-coding RNAs. At present, the majority of the studies have mainly focused on microRNAs. This study represents the first attempt to identify lncRNAs specifically regulated by C/EBPα mutant isoforms in AML. Utilizing AML cell lines modified to produce either wild type or C/EBPα-p30, we have assessed on a global transcriptome level via RNA sequencing the effect these isoforms have on expression of lncRNAs. We were surprised to identify a well-known oncogenic lncRNA, UCA1 [21–24], which was specifically induced by the expression of the C/EBPα-p30 isoform. This was immediately interesting given the fact that the frame-shift mutation causing an over production of the dominant negative p30 isoform is seen in a significant percentage of AML patients [3]. Indeed, we observed a significant up-regulation of UCA1 expression in AML carrying CEBPA mutations. C/EBPα is a strong inducer of differentiation and cell proliferation arrest, however the p30 isoform lacks these abilities due to the absence of the N-terminus [3]. In particular, the p30 isoform lack the transcriptional activation domain that is present in the wild-type C/EBPα and thus it extremely interesting that this isoform is able to specifically induce the transcription of UCA1 lncRNA. Several publications have demonstrated that the p30 isoform is in fact capable of activating specific genes different from the full-length protein [4–7], however, it is still not clear how this is achieved. Further studies are needed to address these issues and to determine its therapeutic implications in AML.

In summary, our work identified, for the first time, an oncogenic long non-coding RNA functioning in concert with the dominant negative C/EBPα-p30 isoform in AML. Since the p30 isoform has transcriptional targets independent of the p42 isoform, it can be speculated that over-expression of the p30 isoform leads to activation of specific genetic programs that contribute to maintain cells in a state of proliferation, with UCA1 might play an integral role. Thus, we propose UCA1 as a novel diagnostic biomarker and a potential therapeutic target for AML with CEBPA mutations.

## MATERIALS AND METHODS

### Cell cultures and reagents

K562 cell lines were maintained in RPMI 1640 medium supplemented with 1 × Penicillin/Streptomicin solution, 1 × L-glutamine and 10% Fetal Bovine Serum. Doxycycline (Dox) was purchased from Sigma and utilized at a concentration of 200 ng/ml, unless differently specified. Stable and inducible CEBPA, CEBPA-P30 and K562 cell lines were produced as previously described [25]. All utilized cell lines were tested for mycoplasma contamination.

### RNA sequencing

Total RNA was extracted using TRIzol (Invitrogen) and the miRNeasy Mini Kit (Qiagen) according to manufacturer instructions. After ribosomal RNA depletion using RiboMinus (Life Technologies), the resulting RNA samples were then used as input for library construction using Illumina TruSeq stranded mRNA sample prep kit (RS-122-2101), as per manufacturer's instructions. RNA libraries were then sequenced on the Illumina Genome Analyzer IIx (86 bp paired-end). For the second sequencing run, total RNA was sent to I.G.A. Applied Genomics Institute (Udine, IT) for library preparation using the Illumina TruSeq stranded mRNA sample prep kit (RS-122-2101) and subjected to sequencing on an Illumina HiSeq 2000 (100 bp paired-end). All RNA-seq data were aligned to hg19 genome using TopHat v2.0.12 with default parameters and assem-bled using Cufflinks 2.2.1 [28] and Gencode v.19 as reference file. We used Cuffdiff v2.2.1 [29] for all differential expression, with a false discovery rate (FDR) of 0.1.

### Data access

The RNA-seq data have been deposited in NCBI's Gene Expression Omnibus (GEO) [40] and are accessible through GEO Series accession number GSE65235.

### RNA extraction and real-time qRT-PCR analysis

Total RNA was extracted using the miRNeasy Kit (Qiagen) according to manufacturer instructions. Reverse transcription to cDNA was performed with the SuperScript VILO cDNA Synthesis Kit (Life Technologies) according to the manufacturer instructions. Quantitative real-time PCR was performed on an Applied Biosystems 7500 Fast Real Time PCR System. Reactions were performed in triplicate using the SYBR green dye detection system and analyzed using 7500 Software v2.0.6 (Applied Biosystems). Relative expression levels of targets were determined using the comparative 2^ΔΔCt^ method. Hypoxanthine-guanine phosphoribosyltransferase (HPRT) mRNA was utilized as a reference.

### UCA1 expression in primary AML samples

Values for UCA1 expression in primary cytogenetically normal AML with CEBPA mutations and wild-type CEBPA (AMLSG cohort; [30]) were obtained from available microarray analysis in GEO (DataSet GDS4278). *P* value was calculated using two-tailed Mann-Whitney *U*-test.

### Immunoblot analysis

40 μg of whole cell extract was separated by 10% SDS-PAGE and electroblotted to nitrocellulose membrane (Protran, S&S). Immunoblots were incubated with antibodies to C/EBPα (sc-61, Santa Cruz Biotechnology), p27^kip1^ (sc-528, Santa Cruz Biotechnology), hnRNPI (sc-56701, Santa Cruz Biotechnology) and GAPDH (sc-25778; Santa Cruz Biotechnology).

### Promoter analysis

The region placed about 400 bp upstream the UCA1 transcription start site was cloned upstream of the FF luciferase in pGL4-Basic Vector (Promega, Madison, WI, USA), giving rise to the UCA1-prom plasmid. Reporter plasmid carrying deletion of the CEBPA binding site (Mut-CEBPA) was obtained by reverse PCR using the oligonucleotides Mut-CEBPA-Fwd and Mut-CEBPA-Rev. 1 μg of each pGL4 derivative plasmid (or an empty vector) and 50 ng of control pRL-TK vector were individually transfected into K562-CTR, K562-CEBPA, and K562-P30. At 12 h post-transfection, half of the cells were treated with dox for transgene expression. After 48 h of induction, cells were harvested and assayed with Dual Luciferase Assay (Promega) according to the manufacturer instructions.

### UCA1 knockdown

UCA1 knockdown was obtained by Mission Lentiviral shRNA clones targeting UCA1 (Sigma-Aldrich, USA). Mission Lentiviral Non-Targeting shRNA clone SHC002 (Sigma-Aldrich, USA) was utilized as control. Lentiviral particles were prepared according to the manufacturer's specifications. Infection of AML cell lines was performed as previously described [41].

### Chromatin immunoprecipitation assay

Protein/DNA cross-linking was obtained by incubating K562 cells for 10 minutes at 37°C in 1% formaldehyde. After sonication, chromatin was immunprecipitated with 5 μg anti C/EBPα antibodies against (sc-61X and sc-9315X, Santa Cruz Biotechnology) as previously described [41]. Immunoprecipitation without specific antibody was performed as a negative control. Negative control amplifications were performed on an intergenic region of chromosome 4. The relative quantity of the immunoprecipitated factor at a locus was estimated using the quantitative threshold method [41].

### RNA immunoprecipitation (RIP)

RIP was performed using 10 μg of hnRNP I antibody (Santa Cruz sc-56701) or isotypic IgGs (Santa Cruz sc-2025) to 30 μl of Protein A/G salmon sperm agarose beads (Millipore) for 2 hr at 4°C. K562 lysates were prepared with 100 μl of RIPA buffer (100 mM NaCl, 20 mM Tris-HCL pH 8, 0.5 5 mM EDTA, 0.5% NP-40) complemented with protease inhibitors and RNasin (Promega); 200 μg of each lysate was used for each RIP assay. Samples were precleared for 1 hr at 4°C with 30 μl of beads, and the supernatant was then resuspended in 600 μl IP buffer (50 mM Tris, pH 7.4, 150 mM NaCl, 1 mM MgCl_2_, 0.05% NP-40) and added to antibody-coated beads for 4 hr at 4°C. Beads were washed with IP buffer five times and split for protein (1/3) and RNA analysis (2/3). Reactions were performed in triplicate using the SYBR green dye detection system and analyzed using serial dilutions of Input for the standard curve setting to determine absolute quantities of each sample. Fold change over background was determined as absolute quantity of IP/IgG.

### Cell proliferation

Cell proliferation and viability was assessed using a Countess Automated Cell Counter (Life Technologies) and Tripan Blue exclusion assay according to manufacturer instruction. For cell cycle analysis, 2 × 10^5^ cells were resuspended in PBS 50% FCS, fixed in 70% ethanol for 24 hours, incubated with 50 μg/mL propidium iodide (Sigma-Aldrich) and 50 units/mL DNase free RNase A (Sigma-Aldrich), and analyzed after 3 hours (10, 000 events) using an Epics XL Cytometer (Beckman Coulter). Data were analyzed with the FCS Express 4 Flow. A minimum of 10,000 events was collected for each sample with flow cytometer (CyAN ADP DAKO) by using Summit 4.3 software for data acquisition and analysis.

### Oligonucleotides utilized in this study

qRT-PCR:

- UCA1: fwd 5′-GACCCTACCCGGTCATTTATAG-3′; rev 5′-CTGATGGGCATGGCTTTATTC-3′.
- HPRT: QuantiTect Primer Assay QT00059066 (Qiagen).

ChIP:

- UCA1 promoter: fwd 5′-TCTCAGGCTGTCCTC-TGGGAAG-3′; rev 5′-TGTAGGCCACCTGGACATA-TATGTG-3′.
- Intergenic control: fwd 5′-TTATCTTGTGGATGTTAGGAAGCA-3′; rev 5′-AATCATGCAGATAATGACCACATC-3′.

UCA1 promoter cloning for luciferase:

- UCA1-Fwd (Xho I): 5′-CCGCTCGAGAGAAATGACCCAGGAGCTGAT-3′;
- UCA1-Rev (Hind III) - 5′-CCCAAGCTTGGTAGGCTGTGGAAAGTTAC-3′;
- Mut-CEBPA-Fwd - 5′-GGGAACTGTCAGGCCTCT-3′
- Mut-CEBPA-Rev- 5′-GTTACAGGGTGATGTGACCT-3′

## SUPPLEMENTARY FIGURES AND TABLES









## References

[1] Tenen DG (2003). Disruption of differentiation in human cancer: AML shows the way. Nat Rev Cancer.

[2] Rosenbauer F, Tenen DG (2007). Transcription factors in myeloid development: balancing differentiation with transformation. Nat Rev Immunol.

[3] Nerlov C (2004). C/EBPalpha mutations in acute myeloid leukaemias. Nat Rev Cancer.

[4] Geletu M, Balkhi MY, Peer Zada AA, Christopeit M, Pulikkan JA, Trivedi AK, Tenen DG, Behre G (2007). Target proteins of C/EBPalphap30 in AML: C/EBPalphap30 enhances sumoylation of C/EBPalphap42 via up-regulation of Ubc9. Blood.

[5] Wang C, Chen X, Wang Y, Gong J, Hu G (2007). C/EBPalphap30 plays transcriptional regulatory roles distinct from C/EBPalphap42. Cell Res.

[6] Pulikkan JA, Dengler V, Peer Zada AA, Kawasaki A, Geletu M, Pasalic Z, Bohlander SK, Ryo A, Tenen DG, Behre G (2010). Elevated PIN1 expression by C/EBPalpha-p30 blocks C/EBPalpha-induced granulocytic differentiation through c-Jun in AML. Leukemia.

[7] Hickey CJ, Schwind S, Radomska HS, Dorrance AM, Santhanam R, Mishra A, Wu YZ, Alachkar H, Maharry K, Nicolet D, Mrózek K, Walker A, Eiring AM (2013). Lenalidomide-mediated enhanced translation of C/EBPα-p30 protein up-regulates expression of the antileukemic microRNA-181a in acute myeloid leukemia. Blood.

[8] Pabst T, Mueller BU, Zhang P, Radomska HS, Narravula S, Schnittger S, Behre G, Hiddemann W, Tenen DG (2001). Dominant-negative mutations of CEBPA, encoding CCAAT/enhancer binding protein-alpha (C/EBPalpha), in acute myeloid leukemia. Nat Genet.

[9] Pabst T, Mueller BU (2007). Transcriptional dysregulation during myeloid transformation in AML. Oncogene.

[10] Kirstetter P, Schuster MB, Bereshchenko O, Moore S, Dvinge H, Kurz E, Theilgaard-Mönch K, Månsson R, Pedersen TA, Pabst T, Schrock E, Porse BT, Jacobsen SE (2008). Modeling of C/EBPalpha mutant acute myeloid leukemia reveals a common expression signature of committed myeloid leukemia-initiating cells. Cancer Cell.

[11] Katzerke C, Madan V, Gerloff D, Bräuer-Hartmann D, Hartmann JU, Wurm AA, Müller-Tidow C, Schnittger S, Tenen DG, Niederwieser D, Behre G (2013). Transcription factor C/EBPα-induced microRNA-30c inactivates Notch1 during granulopoiesis and is downregulated in acute myeloid leukemia. Blood.

[12] Pulikkan JA, Peramangalam PS, Dengler V, Ho PA, Preudhomme C, Meshinchi S, Christopeit M, Nibourel O, Müller-Tidow C, Bohlander SK, Tenen DG, Behre G (2010). C/EBPα regulated microRNA-34a targets E2F3 during granulopoiesis and is down-regulated in AML with CEBPA mutations. Blood.

[13] Eyholzer M, Schmid S, Wilkens L, Mueller BU, Pabst T (2010). The tumour-suppressive miR-29a/b1 cluster is regulated by CEBPA and blocked in human AML. Br J Cancer.

[14] Fazi F, Rosa A, Fatica A, Gelmetti V, De Marchis ML, Nervi C, Bozzoni I (2005). A minicircuitry comprised of microRNA-223 and transcription factors NFI-A and C/EBPalpha regulates human granulopoiesis. Cell.

[15] Zhang H, Chen Z, Wang X, Huang Z, He Z, Chen Y (2013). Long non-coding RNA: a new player in cancer. J Hematol Oncol.

[16] Batista PJ, Chang HY (2013). Long noncoding RNAs: cellular address codes in development and disease. Cell.

[17] Fatica A, Bozzoni I (2014). Long non-coding RNAs: new players in cell differentiation and development. Nat Rev Genet.

[18] Morlando M, Ballarino M, Fatica A (2015). Long Non-Coding RNAs: New Players in Hematopoiesis and Leukemia. Front Med.

[19] Guo G, Kang Q, Chen Q, Chen Z, Wang J, Tan L, Chen JL (2014). High expression of long non-coding RNA H19 is required for efficient tumorigenesis induced by Bcr-Abl oncogene. FEBS Lett.

[20] Guo G, Kang Q, Zhu X, Chen Q, Wang X, Chen Y, Ouyang J, Zhang L, Tan H, Chen R, Huang S, Chen JL (2014). A long noncoding RNA critically regulates Bcr-Abl-mediated cellular transformation by acting as a competitive endogenous RNA. Oncogene.

[21] Fan Y, Shen B, Tan M, Mu X, Qin Y, Zhang F, Liu Y (2014). Long non-coding RNA UCA1 increases chemoresistance of bladder cancer cells by regulating Wnt signaling. FEBS J.

[22] Han Y, Yang YN, Yuan HH, Zhang TT, Sui H, Wei XL, Liu L, Huang P, Zhang WJ, Bai YX (2014). UCA1, a long non-coding RNA up-regulated in colorectal cancer influences cell proliferation, apoptosis and cell cycle distribution. Pathology.

[23] Huang J, Zhou N, Watabe K, Lu Z, Wu F, Xu M, Mo YY (2014). Long non-coding RNA UCA1 promotes breast tumor growth by suppression of p27 (Kip1). Cell Death Dis.

[24] Xue M, Li X, Li Z, Chen W (2014). Urothelial carcinoma associated 1 is a hypoxia-inducible factor-1α-targeted long noncoding RNA that enhances hypoxic bladder cancer cell proliferation, migration, and invasion. Tumour Biol.

[25] Hughes JM, Salvatori B, Giorgi FM, Bozzoni I, Fatica A (2014). CEBPA-regulated lncRNAs, new players in the study of acute myeloid leukemia. J Hematol Oncol.

[26] Tavor S, Park DJ, Gery S, Vuong PT, Gombart AF, Koeffler HP (2003). Restoration of C/EBPalpha expression in a BCR-ABL+ cell line induces terminal granulocytic differentiation. J Biol Chem.

[27] Ferrari-Amorotti G, Keeshan K, Zattoni M, Guerzoni C, Iotti G, Cattelani S, Donato NJ, Calabretta B (2006). Leukemogenesis induced by wild-type and STI571-resistant BCR/ABL is potently suppressed by C/EBPalpha. Blood.

[28] Trapnell C, Roberts A, Goff L, Pertea G, Kim D, Kelley DR, Pimentel H, Salzberg SL, Rinn JL, Pachter L (2012). Differential gene and transcript expression analysis of RNA-seq experiments with TopHat and Cufflinks. Nat Protoc.

[29] Trapnell C, Hendrickson DG, Sauvageau M, Goff L, Rinn JL, Pachter L (2013). Differential analysis of gene regulation at transcript resolution with RNA-seq. Nat Biotechnol.

[30] Taskesen E, Bullinger L, Corbacioglu A, Sanders MA, Erpelinck CA, Wouters BJ, van der Poel-van de Luytgaarde SC, Damm F, Krauter J, Ganser A, Schlenk RF, Löwenberg B, Delwel R (2011). Prognostic impact, concurrent genetic mutations, and gene expression features of AML with CEBPA mutations in a cohort of 1182 cytogenetically normal AML patients: further evidence for CEBPA double mutant AML as a distinctive disease entity. Blood.

[31] Garzon R, Volinia S, Papaioannou D, Nicolet D, Kohlschmidt J, Yan PS, Mrózek K, Bucci D, Carroll AJ, Baer MR, Wetzler M, Carter TH, Powell BL (2014). Expression and prognostic impact of lncRNAs in acute myeloid leukemia. Proc Natl Acad Sci U S A.

[32] Xue M, Li X, Wu W, Zhang S, Wu S, Li Z, Chen W (2014). Upregulation of long non-coding RNA urothelial carcinoma associated 1 by CCAAT/enhancer binding protein α contributes to bladder cancer cell growth and reduced apoptosis. Oncol Rep.

[33] Tsukada J, Yoshida Y, Kominato Y, Auron PE (2011). The CCAAT/enhancer (C/EBP) family of basic-leucine zipper (bZIP) transcription factors is a multifaceted highly-regulated system for gene regulation. Cytokine.

[34] Wang F, Li X, Xie X, Zhao L, Chen W (2008). UCA1, a non-protein-coding RNA up-regulated in bladder carcinoma and embryo, influencing cell growth and promoting invasion. FEBS Lett.

[35] Wang Y, Chen W, Yang C, Wu W, Wu S, Qin X, Li X (2012). Long non-coding RNA UCA1a(CUDR) promotes proliferation and tumorigenesis of bladder cancer. Int J Oncol.

[36] Tay Y, Kats L, Salmena L, Weiss D, Tan SM, Ala U, Karreth F, Poliseno L, Provero P, Di Cunto F, Lieberman J, Rigoutsos I, Pandolfi PP (2011). Coding-independent regulation of the tumor suppressor PTEN by competing endogenous mRNAs. Cell.

[37] Tseng YY, Moriarity BS, Gong W, Akiyama R, Tiwari A, Kawakami H, Ronning P, Reuland B, Guenther K, Beadnell TC, Essig J, Otto GM, O'Sullivan MG (2014). PVT1 dependence in cancerwith MYC copy-number increase. Nature.

[38] Trimarchi T, Bilal E, Ntziachristos P, Fabbri G, Dalla-Favera R, Tsirigos A, Aifantis I (2014). Genome-wide mapping and characterization of Notch-regulated long noncoding RNAs in acute leukemia. Cell.

[39] Gómez-Maldonado L, Tiana M, Roche O, Prado-Cabrero A, Jensen L, Fernandez-Barral A, Guijarro-Muñoz I, Favaro E, Moreno-Bueno G, Sanz L, Aragones J, Harris A, Volpert O (2014). EFNA3 long noncoding RNAs induced by hypoxia promote metastatic dissemination. Oncogene.

[40] Barrett T, Wilhite SE, Ledoux P, Evangelista C, Kim IF, Tomashevsky M, Marshall KA, Phillippy KH, Sherman PM, Holko M, Yefanov A, Lee H, Zhang N (2013). NCBI, GEO: archive for functional genomics data sets–update. Nucleic Acids Res.

[41] Salvatori B, Iosue I, Djodji Damas N, Mangiavacchi A, Chiaretti S, Messina M, Padula F, Guarini A, Bozzoni I, Fazi F, Fatica A (2011). Critical Role of c-Myc in Acute Myeloid Leukemia Involving Direct Regulation of miR-26a and Histone Methyltransferase EZH2. Genes Cancer.

