# Supplementary material for: C/EBPα-p30 protein induces expression of the oncogenic long non-coding RNA UCA1 in acute myeloid leukemia
Source: Oncotarget. 2015 May 25;6(21):18534–44. doi: 10.18632/oncotarget.4069 (PMC4621908; doi:10.18632/oncotarget.4069)
Supplement: Supplementary file 1 [file oncotarget-06-18534-s001.pdf]

SUPPLEMENTARY FIGURES AND TABLES

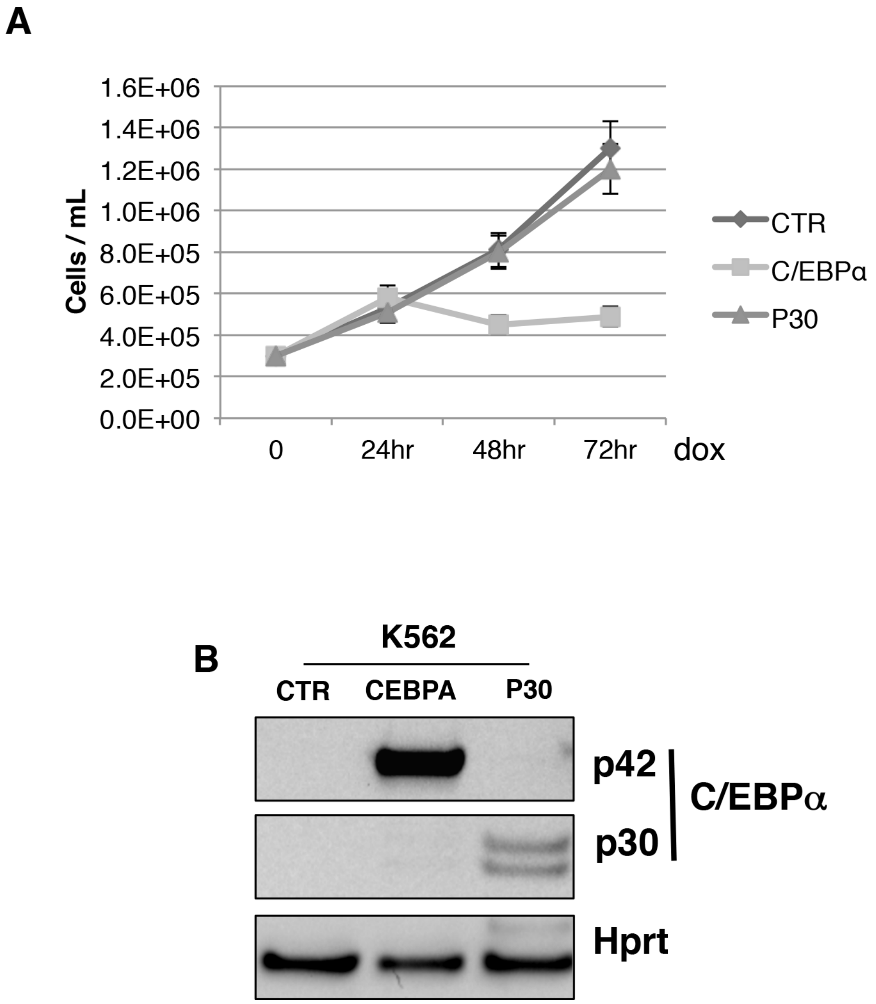

Supplementary Figure S1: Effects of C/EBPα and p30 expression in K562 cells. A. Growth curve of K562 cells containing CTR, CEBPA and P30 expression cassette, respectively, after induction with Doxycyline (dox). **B.** Western blot after 48 hrs of dox induction confirms the expression of C/EBPα isoforms (p42 and p30) the CEBPA stable cell line, and not in the CTR empty vector cell line.

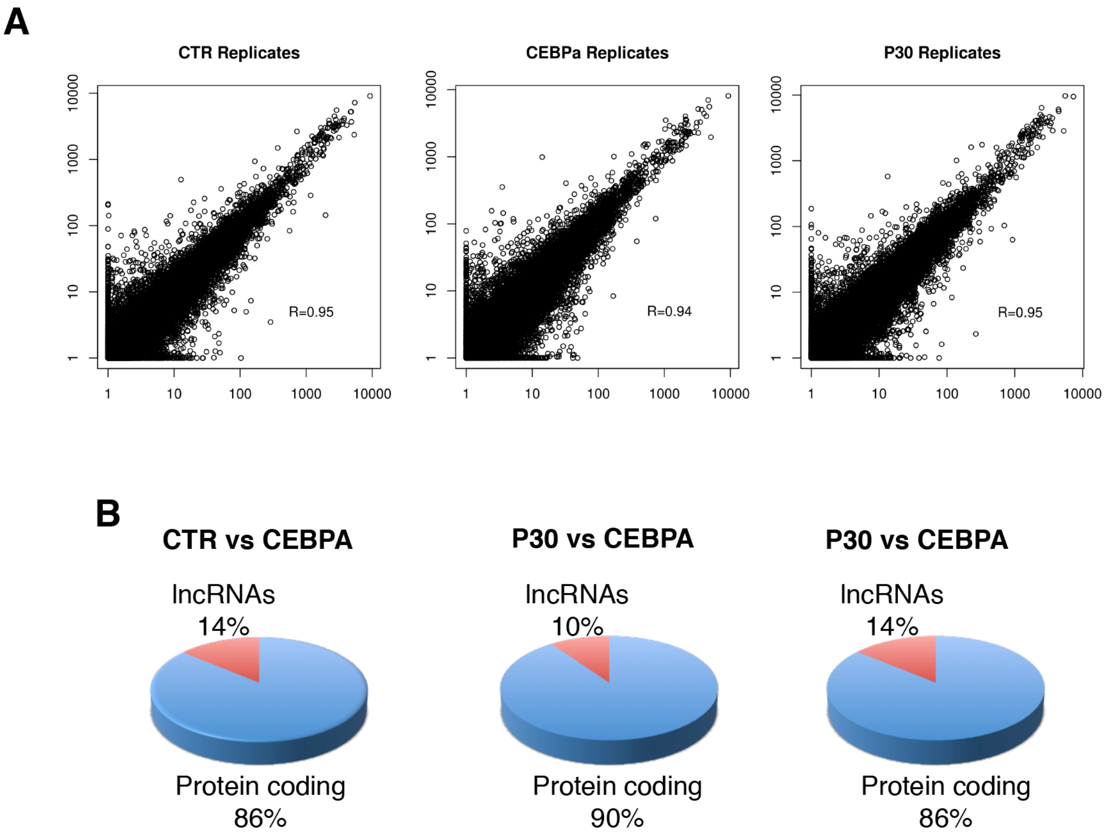

C

Biotype	CTR vs CEBPA	CTR vs P30	CEBPA vs P30
3-Prime Overlapping ncRNA	0	0	0
Antisense	28	2	21
lincRNA	79	1	72
misc RNA	1	0	0
Polymorphic Pseudogene	0	0	1
Processed Pseudogene	0	0	0
Processed Transcript	5	2	5
Protein Coding	1049	55	949
Pseudogene	51	2	53
Sense Intronic	2	0	0
Sense Overlapping	4	1	4

Supplementary Figure S2: RNA-seq data. A. Correlation analysis among replicate sequencing runs confirms a near 95% correlation between replicates. **B.** The charts show the distribution of significantly up-regulated genes between protein coding and lncRNAs. **C.** Biotypes of significantly up-regulated genes.

Supplementary Table S1: RNAs with differential expression between CTR and CEBPA samples

Supplementary Table S2: RNAs with differential expression between CEBPA and P30 samples

Supplementary Table S3: RNAs with differential expression between CTR and P30 samples
